# Disease Severity and Effective Parasite Multiplication Rate in Falciparum Malaria

**DOI:** 10.1093/ofid/ofx169

**Published:** 2017-12-20

**Authors:** Hugh W Kingston, Aniruddha Ghose, Katherine Plewes, Haruhiko Ishioka, Stije J Leopold, Richard J Maude, Sanjib Paul, Benjamas Intharabut, Kamorat Silamut, Charles Woodrow, Nicholas P J Day, Kesinee Chotivanich, Nicholas M Anstey, Amir Hossain, Nicholas J White, Arjen M Dondorp

**Affiliations:** 1 Mahidol Oxford Tropical Medicine Research Unit, Faculty of Tropical Medicine, Mahidol University, Bangkok, Thailand; 2 Global and Tropical Health Division, Menzies School of Health Research and Charles Darwin University, Darwin, Northern Territory, Australia; 3 Chittagong Medical College, Chittagong, Bangladesh; 4 Centre for Tropical Medicine and Global Health, Nuffield Department of Clinical Medicine, Churchill Hospital, Oxford, United Kingdom

**Keywords:** malaria severity, parasite multiplication rate, parasite biomass, Plasmodium falciparum, *PfHRP2*

## Abstract

Patients presenting with severe falciparum malaria in a Bangladeshi tertiary hospital had higher total parasite burden, estimated by parasitemia and plasma PfHRP2, than uncomplicated malaria patients despite shorter fever duration. This suggests that higher parasite multiplication rates (PMR) contribute to causing the higher biomass found in severe disease. Compared with patients without a history of previous malaria, patients with previous malaria carried a lower parasite biomass with similar fever duration at presentation, suggesting that host immunity reduces the PMR.

Why some patients develop severe falciparum malaria and others do not is poorly understood [1, 2]. One central factor in determining disease severity in many infectious diseases is the number of organisms in the body. In malaria, the parasite biomass is determined by the number of merozoites emerging from the liver at the end of preerythrocytic development (around 10^5^), the effective parasite multiplication rate (PMR) (the average number of schizont progeny that complete the 48-hour asexual cycle), and the duration of blood stage infection [2]. The risk of developing severe malaria increases with parasite biomass [3]. Controlled human malaria infection (CHMI) is safe in malaria-naïve subjects because the infections are terminated after only a few asexual cycles at a low biomass, typically before or when parasitemia is detected by microscopy [4]. CHMI studies in people with previous malaria found that PMRs are lower (2 vs 8), indicating that immunity reduces parasite multiplication [5]. In both malaria therapy and experimental malaria, PMR before antimalarial treatment varied between patients [6], and in some patients, parasitemia peaked and then declined without treatment, indicating that the PMR has fallen below 1 [7]. Several factors other than immunity affect parasite multiplication, including hemoglobinopathies, fever, and the infecting parasite strain [8]. Thus, either variation in PMR or duration of blood stage infection before treatment may result in a higher parasite biomass at presentation, and thus an increased risk of developing severe disease. While it is well established that delayed antimalarial treatment may result in severe disease, the role of variation in effective PMR has been uncertain.

## METHODS

### Patients and Measurements

Data from adult patients with falciparum malaria enrolled in prospective studies conducted between 2003–2014 at Chittagong Medical College (CMCH) in Chittagong, Bangladesh, were analyzed. Informed consent was obtained from all patients, or their relatives if patients lacked capacity. Studies were approved by the local ethics committee and the Oxford Tropical Research Ethics Committee. Patients were enrolled if they had a blood slide positive for asexual *Plasmodium falciparum* parasites. Severe malaria was defined exactly as described previously [9]. The following patients were excluded from the analysis: patients who had received more than 12 hours of treatment with an effective antimalarial (as pretreatment would affect fever duration, parasite count, and *P. falciparum* histidine-rich protein [PfHRP2]), patients age <16 years (as there is evidence that the development of immunity in children is different than in adults [1]), and patients without a plasma PfHRP2 measurement. History and physical examination findings were recorded at enrollment. Specifically, patients or their attendants were asked about the duration of fever before enrollment, if they had had malaria before, and how many malaria episodes they had suffered previously. Plasma PfHRP2 at enrollment was measured by enzyme-linked immunosorvent assay in ethylenediaminetetraacetic acid (EDTA) or heparin plasma, as reported previously [9].

### Statistics

Continuous variables were compared between groups using the Mann-Whitney U-test. Correlations were assessed using Spearman’s rank correlation. Nonlinear relationships were assessed using the method of fractional polynomials. An alpha of <.05 was used as the threshold for statistical significance. All analysis was performed using Stata version 14 (StataCorp, College station, TX).

## RESULTS

### Relationship Between Parasite Biomass Markers, Duration of Illness, and Malaria Severity

**Figure 1.**
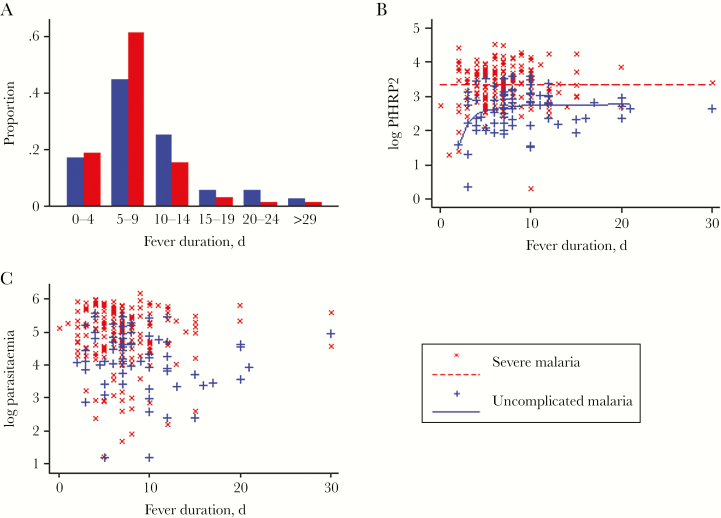
Fever duration in uncomplicated and severe malaria, data for fever duration <30 days shown. Logarithms are base 10. (A) Distribution of fever duration in uncomplicated and severe malaria. (B) Relationship between log PfHRP2 (ng/mL) and fever duration in adults with uncomplicated or severe malaria. Lines indicate best fit (uncomplicated malaria, first-order fractional polynomial, severe malaria fever is not predictive). (C) Relationship between log parasitemia (μL) and fever duration in adults with uncomplicated or severe malaria.

In total, 203 patients with severe and 77 with uncomplicated falciparum malaria were studied. Sixty-eight (33%) of the patients with severe malaria died. Median fever duration was 7 days (interquartile range [IQR], 5–9 days) in severe and 7.5 days (IQR, 6–10.5 days) in uncomplicated malaria (*P* = .01) (Table 1, Figure 1A). The odds of severe malaria decreased with longer fever duration (odds ratio [OR] per day, 0.93; 95% confidence interval [CI], 0.87–0.98), and in patients with severe malaria there was a borderline trend for the odds of death to decrease with longer fever duration (OR, 0.92; 95% CI, 0.84–1.01). Plasma PfHRP2 and parasitemia were weakly correlated (*rho*, 0.27, n = 272, *P* < .0001) and were both significantly higher in the patients with severe malaria (Table 1). Parasitemia and PfHRP2 did not correlate with fever duration in either severe or uncomplicated malaria (*P* > .05) (Figure 1, B and C). The relationship between plasma PfHRP2 and fever duration appeared nonlinear in uncomplicated malaria, and a first-degree fractional polynomial (–2) provided the best fit (*P* < .01 vs constant only or linear untransformed model), indicating an increase followed by a plateau of PfHRP2 with fever duration (Figure 1B). No significant nonlinear relationships were found for parasitaemia and fever duration.

**Table 1.** Baseline Characteristics and Outcome

	Uncomplicated Malaria	Severe Malaria	*P* Value
	(n = 77)	(n = 203)	
Age, years	25 (20 to 40)	32 (24 to 45)	.02
Fever duration, days	7.5 (6 to 10.5)	7 (5 to 9)	.01
Hematocrit, %	31 (25 to 37)	28 (22 to 35)	.03
Plasma PfHRP2, ng/mL	504 (171.2 to 1036)	2565 (947 to 5278)	<.001
Parasitemia, μL	18 870 (6894 to 62409)	138 380 (34 670 to 339 660)	<.001
Base deficit, mmol/l	–1 (–3 to 1)	–7 (–11 to –4)	<.001
BUN, mg/dl	15 (11 to 25)	41.2 (24 to 70)	<.001
GCS	15 (15 to 15)	9 (7 to 12)	<.001
Sex, % male	74	69	.41
Previous malaria, %	31	16	.009
Coma, %	NA	70	NA
Hyperparasitemia, %	NA	28	NA
Acidosis, %	NA	34	NA
Acute renal failure, %	NA	24	NA
Anemia, %	13	21	.15
Jaundice, %	33	38	.46
Died, %	NA	33	NA

Brackets contain interquartile range. Dichotomous variables are defined as previously used [9]. Uncomplicated malaria parasitemia (n = 69). Abbreviations: PfHRP2, *Plasmodium falciparum* histidine-rich protein 1; BUN, blood urea nitrogen; GCS, Glasgow Coma Score; NA, not applicable; n, number of patients for whom data was available.

### Relationship Between Parasite Biomass Markers and Prior Malaria Episodes

An episode of previous malaria was associated with significant protection from severe malaria (OR, 0.42; 95% CI, 0.22–0.81) but not death (OR, 0.63; 95% CI, 0.25–1.59), but this effect was not independent of a marker of biomass (reflected by log(PfHRP2) in plasma). Overall, an episode of previous malaria was associated with lower plasma PfHRP2 (median, 860 vs 2110 ng/mL, *P* < .001) but was not associated with shorter fever duration (median, 7 days both groups, *P* = .99). Peripheral blood parasitemia was lower in patients with previous malaria compared with those presenting with a first episode (median, 37 771 vs 100 388/μL, respectively, *P* = .001). Stratifying by disease severity, this difference was significant in patients with severe malaria (median, 41 910 vs 149 450/μL, respectively, *P* = .009) but not uncomplicated malaria (17 738 vs 24 927/μL, respectively, *P* = .7). Age but not sex was a significant predictor of malaria severity (Table 1). Age did not correlate with fever duration, pfHRP2, or parasitaemia overall or within the severe or uncomplicated malaria groups.

In a multivariate logistic regression model for malaria severity including prior malaria, log pfHRP2, fever duration, and log age, only log pfHRP2 (OR, 8.86; 95% CI, 4.49–17.48), fever duration (OR, 0.89; 95% CI, 0.82–0.97), and age (OR, 1.04; 95% CI, 1.01–1.07) remained significant predictors (McFadden’s *R*^2^, 0.32).

## DISCUSSION

Early diagnosis and treatment of falciparum malaria prevent severe disease and death. It is often assumed, therefore, that patients develop severe malaria because of delays in treatment. However, this study, conducted in an area of low seasonal transmission, suggests that a more fulminant disease process is present in severe disease. Patients with severe disease had higher plasma PfHRP2 and parasitemia yet slightly shorter durations of illness compared with adults presenting with uncomplicated falciparum malaria. A history of previous malaria was associated with a lower odds ratio for severe disease and lower estimated total parasite burden, but not a shorter duration of fever.

These observations indicating a higher PMR in severe disease are consistent with a previous study from Asia in which the PMR measured ex vivo was higher for parasites from severe as opposed to uncomplicated malaria [8]. However, these findings were not replicated in a study in African children with falciparum malaria in an area where transmission is much higher and immune recognition was presumably the main determinant of malaria severity and outweighed any differences in intrinsic parasite virulence [10]. The lack of correlation between plasma PfHRP2 and fever duration in severe malaria and the weak nonlinear relationship in uncomplicated malaria suggest variations in PMR within these groups. The data also suggest a decrease in PMR with duration of fever as a nonlinear relationship was observed between fever duration and plasma PfHRP2 in patients with uncomplicated malaria (Figure 1B). This is concordant with malariatherapy data indicating that parasitemia plateaus and then falls during the course of infection, consistent with a reduction in the PMR [7].

In Bangladesh, malaria transmission is seasonal [11]. Previous exposure to malaria was associated with a reduced risk of severe disease and reduced parasite biomass markers. This suggests augmentation of host-defense mechanisms after even a single infection [1]. CHMI studies in adults with a history of previous malaria have reported a reduction in PMR during the prepatent phase [5]. Similarly, in adults undergoing malaria therapy for neurosyphilis, peaks of parasitemia were lower in second and subsequent infections [12]. Previous exposure to malaria has also been associated with protection from severe malaria in travelers [13]. These observations are all consistent with host-defense responses such as antibody-mediated clearance of merozoites or infected red cells, limiting expansion of the biomass and hence reducing the risk of severe disease. Additional factors such as parasite and host genotype may also affect PMR, which merits further investigation. Variation in the fever threshold could also influence the relationship between parasite biomass and fever duration; the fever threshold rises with more frequent exposure to malaria [14]. However, this is unlikely to explain the findings of the present study as no difference in fever duration was observed according to exposure status. As in previous studies [13], age was a significant predictor of malaria severity. There was no correlation between age and pfHRP2 or parasitaemia, suggesting that a biomass-independent mechanism underlies the relationship with severity.

Limitations of this study include that patients or their attendants may report fever duration inaccurately, which could obscure a relationship with biomass. Consistent with this being reported relatively accurately, a previous study at this hospital observed the expected association between the duration of symptoms prior to admission and the distance from the hospital [15]. Patients may report “prior malaria” inaccurately. The relative importance of delayed presentation vs low PMR may be different in different settings. Only data from patients presenting to a tertiary referral center have been analyzed here.

In summary, intrahost parasite dynamics are different in adults with severe and uncomplicated malaria—patients with severe disease appear to have had a higher PMR and generally present with a larger biomass than those with uncomplicated malaria.
